# Editorial: Deciphering host-pathogen interactions in tuberculosis: implications for diagnostics and therapeutics

**DOI:** 10.3389/fimmu.2026.1809254

**Published:** 2026-04-14

**Authors:** Mariana Araujo-Pereira, Selvakumar Subbian

**Affiliations:** 1 Goncalo Moniz Institute (IGM), Salvador, Brazil; 2 Public Health Research Institute, New Jersey Medical School, Rutgers University, Newark, NJ, United States

**Keywords:** co-morbidities, diagnosis, genetics, immune response, mycobacterium, preclinical models, tuberculosis, vaccines

## Introduction

Tuberculosis (TB), caused by Mycobacterium tuberculosis (Mtb) persists as serious global health concern with more than a million deaths and 10 million new cases in 2024 (1). Recent TB research continues to evolve at the intersection of diagnostics, host-pathogen interactions, host immunity, as well as clinical complexity and therapeutic innovations. This Research Topic contains original and review articles from studies that collectively illustrate how advances in molecular technologies, immunological insight, and population-level analyses are reshaping the understanding and management of TB across diverse patient populations, ranging from neonates and immunocompromised adults to large longitudinal cohorts. Together, these reports underscore a shift toward precision approaches that integrate TB diagnosis with host-response profiling and clinically actionable prognostic tools.

Progress in TB diagnostics is exemplified by the work of Li et al., who evaluated metagenomic Mtb detection across tissue, blood, and sputum samples in patients with suspected infection, many of whom were living with the human immunodeficiency virus (HIV) infection. By directly sequencing Mtb DNA without prior assumptions, this approach achieved markedly higher sensitivity and specificity than conventional diagnosis such as histopathology, culture, or targeted PCR. Beyond accuracy, metagenomics provided clues on disease distribution, highlighting lymph nodes as a frequent site of Mtb infection and revealed a surprisingly high burden of non-tuberculous mycobacteria (NTM) in HIV-positive individuals. These findings reinforce the value of unbiased diagnostics in immunocompromised hosts, where atypical disease presentations and mixed infections are common, while also pointing to the need for multicenter validation and economic evaluation of newer diagnostic tests before routine implementation.

Epigenetic biomarkers provide a complementary, host-centered diagnostic approach. Wang et al. investigated host DNA methylation patterns in key “Wingless related integration site” (Wnt) signaling genes in peripheral blood and demonstrated consistent hypomethylation in patients with pulmonary TB compared with healthy controls. Certain methylation signatures correlated with clinical characteristics such as drug resistance, fever, and treatment-related liver injury, suggesting that epigenetic alterations may reflect both TB disease susceptibility and host stress responses. Although diagnostic performance of proposed epigenetic markers was modest, especially at the single-gene level, the study highlights the feasibility of noninvasive blood-based assays that capture immune-pathway perturbations, which can further be explored in larger and more diverse cohorts.

Small non-coding RNAs further bridge diagnostics and pathogenesis in TB. Jahan et al., demonstrated that exosomal miR-17-5p is upregulated during mycobacterial infection and directly suppresses Mitogen Activated Protein Kinase Kinase Kinase 2 (MAP3K2), leading to reduced MAPK signaling, diminished inflammatory cytokine production, and impaired antimicrobial effector functions. This mechanism facilitates intracellular mycobacterial survival, positioning miR-17-5p as both a mediator of immune evasion and a circulating biomarker to distinguish people with active disease from healthy subjects. In contrast, Xu et al. identified miR-107–enriched exosomes as a host-protective signal in TB. These vesicles enhance reactive oxygen species (ROS) generation, activate canonical Wnt signaling, and promote autophagy, resulting in suppressed intracellular bacterial growth. Importantly, intranasal administration of miR-107–rich exosomes reduced lung bacterial burden and inflammation *in vivo*, highlighting a rare convergence of diagnostic potential and therapeutic applicability within the same molecular system.

While advanced diagnostics illuminate disease, host genetic and immunological factors determine TB susceptibility and treatment outcome. Hu et al. explored a functional variant in the Neutrophil Cytosolic Factor 1 (NCF1) gene, previously linked to autoimmune disease, and identified a sex-specific protective effect against TB in women carrying the AA genotype. Reduced neutrophil counts and diminished ROS production were proposed as mechanisms that limit neutrophil-driven tissue damage and necrotic pathology while preserving pathogen control. This work exemplifies evolutionary trade-offs in immune defense and underscores the importance of considering sex as a biological variable in genetic association studies in the context of infectious diseases.

Sex-specific immune regulation was also central to the study by Hertz et al., who demonstrated that B cell–derived interleukin-10 (IL-10) promotes TB susceptibility in a mouse model, with effects most pronounced in males. Deletion of IL-10 specifically in B cells resulted in delayed disease progression, lower lung bacterial loads, and prolonged survival, accompanied by increased interferon gamma (IFN-γ) production and remodeling of pulmonary immune cell populations. Transcriptomic analyses revealed broad sex-dependent immune reprogramming, implicating pathways such as Janus Kinase (JAK)–Signal Transducer and Activator of Transcription (STAT) signaling. These findings reposition B cells as key regulators of TB immunity and suggest that immunomodulatory interventions may require sex-adapted strategies to achieve maximal benefit.

Functional immune profiling in human populations complements these mechanistic insights. Oo et al. characterized peripheral blood mononuclear cells (PBMCs) from individuals with active TB disease, latent infection, and healthy controls in an endemic setting. Using mycobacterial growth inhibition assays alongside flow cytometry, the study showed that cells from patients with active disease exhibited enhanced capacity to restrict mycobacterial growth, associated with increased natural killer (NK) cells and robust TNF responses from CD4^+^ and CD8^+^ T cells. Specific cytokine profiles correlated inversely with bacterial replication, suggesting that qualitative features of immune responses may serve as correlates of protection against TB and inform vaccine development or patient stratification.

In another study, Zhu et al. report distinct gene expression profile of CD3+/IFNγ+ T cells from the PBMCs of healthy individuals when stimulated with BCG vaccine or Mtb. In these cells, BCG stimulation upregulated IL-2 resulted in a positive feedback loop that amplifies the cell population expressing IFNγ. This study highlights the role of IL2 by CD3+/IFNγ+ T cells in anti-TB immunity.

Nutritional and metabolic factors further modulate immune responses to TB. Moideen et al. documented significant alterations in serum mineral concentrations among patients with pulmonary disease, including elevated copper levels and increased copper-to-zinc and copper-to-selenium ratios, alongside reduced zinc and selenium. These imbalances correlated with both pro- and anti-inflammatory cytokines and normalized following anti-tuberculous therapy, implicating mineral ratios as integrative biomarkers of systemic inflammation. The findings highlight the potential of nutritional interventions as adjuncts to therapy, while also cautioning that causal relationships and optimal supplementation strategies require longitudinal validation.

Beyond host susceptibility, therapeutic innovation increasingly emphasizes host-directed strategies. Sabeel et al. investigated the immunomodulatory effects of atorvastatin in infected PBMCs and demonstrated dose-dependent suppression of intracellular mycobacterial growth. Mechanistically, atorvastatin enhanced phagosome maturation, induced autophagy, and promoted apoptosis through pathways linked to inhibition of the mevalonate cascade. Selective reduction of IL-1β without broad cytokine suppression suggested a targeted anti-inflammatory effect. These findings support the repurposing of statins as adjunctive host-directed therapies and provide a mechanistic rationale for clinical trials integrating immunological endpoints.

Pathogen-centered biology remains equally critical for long-term control. Zhang et al. integrated the available evidence on the PE/PPE protein family of Mtb, which plays central roles in immune modulation, nutrient acquisition, and virulence. These proteins interact with host receptors, disrupt phagosome maturation, and influence cytokine networks and cell death pathways. Their immunogenic properties have already been leveraged in vaccine candidates showing promise in clinical trials, as well as in diagnostic assays distinguishing latent from active disease. However, functional redundancy and antigenic variability pose challenges, emphasizing the need for high-resolution structural and systems-level analyses to guide rational vaccine and diagnostic design.

Clinical practice in TB often reveals complexities that transcend experimental models. Tang et al. reported a rare neonatal case of concurrent TB and pertussis in a 47-day-old infant, diagnosed through comprehensive molecular testing of bronchoalveolar lavage fluid (BAL). Successful management of this case required carefully balanced antimicrobial regimens and vigilant monitoring for toxicity. This clinical presentation underscores the importance of broad diagnostic evaluation in infants with atypical respiratory disease and highlights the vulnerability of early life to overlapping infections.

Similarly, Tian et al. described the first documented coexistence of papillary renal cell carcinoma with ipsilateral renal TB. Radiologic findings suggested malignancy, but definitive diagnosis relied on postoperative histopathology and molecular testing. This case illustrates how renal TB can mimic or coexist with cancer, complicating diagnosis and management, and reinforces the indispensable role of tissue-based confirmation of disease pathology when imaging is inconclusive.

At the population level, prognostic tools derived from routine laboratory data offer practical value. Ji et al. analyzed a large cohort of TB patients and demonstrated that elevated baseline inflammatory indices, such as neutrophil-to-lymphocyte and systemic immune-inflammation ratios, were independently associated with higher risks of treatment failure and mortality. Incorporating these indices into predictive models significantly improved prognostic performance beyond conventional clinical variables. Despite limitations inherent to retrospective design, the study highlights how simple, widely available measures can inform risk stratification and guide clinical decision-making.

**Figure 1:**
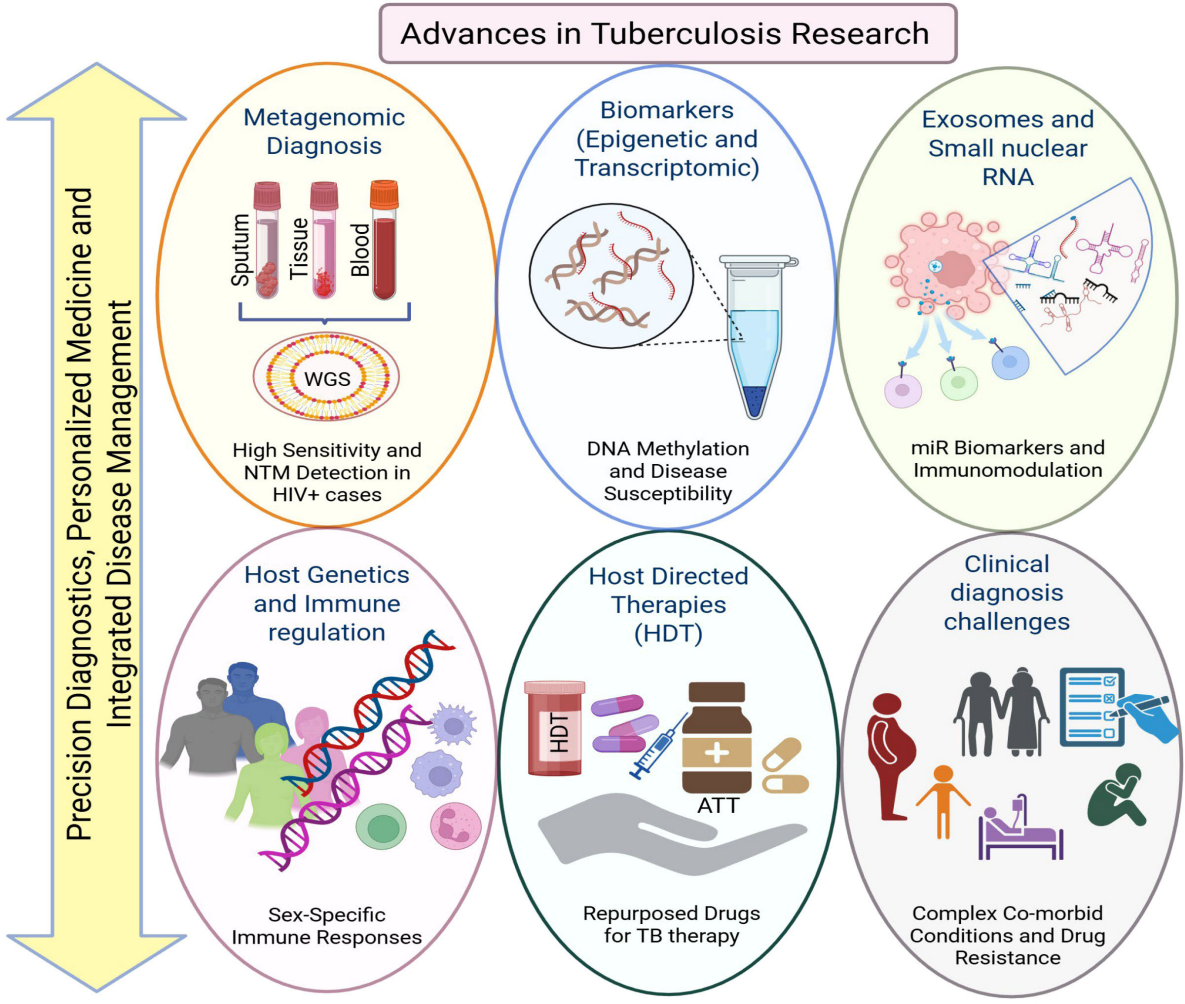
Summary of key findings from studies reported in this Research Topic, which collectively advance TB research. The articles highlight metagenomic sequencing for improved pathogen detection, blood-based epigenetic and microRNA biomarkers of host immune perturbation, sex-specific genetic and immunological determinants of susceptibility, host-directed therapeutic strategies that enhance antimicrobial pathways, and clinical challenges arising from atypical co-infections and complex presentations. Together, these integrated insights into host–pathogen interactions inform emerging precision approaches for TB diagnosis, treatment, and prognosis. Image created with BioRender.com.

Taken together, articles in this collecton highlight the advances in sequencing, epigenetics, and exosomal biology that are expanding TB diagnostic and therapeutic horizons, while immunological and population-level insights refine understanding of risk and treatment outcome (**Figure 1**). The collective challenge ahead lies in integrating these discoveries into coherent, scalable strategies that improve diagnosis, personalize therapy, such as host-directed therapies (HDT), and ultimately reduce the global TB burden (2).
